# Antiproliferative activity of Tamoxifen, Vitamin D3 and their concomitant treatment

**DOI:** 10.17179/excli2021-3989

**Published:** 2021-09-21

**Authors:** Derya Yetkin, Ebru Balli, Furkan Ayaz

**Affiliations:** 1 Mersin University, Advanced Technology Education Research and Application Center, 33110, Mersin, Turkey; 2 Mersin University, Department of Histology and Embryology, 33110 Mersin, Turkey; 3 Mersin University, Department of Biotechnology, Faculty of Arts and Science, 33110, Mersin, Turkey

**Keywords:** breast cancer, Tamoxifen, Vitamin D3, cell proliferation, apoptosis, cell cycle

## Abstract

Breast cancer stands out as the most common cancer type among women throughout the world. Especially for the estrogen receptor alpha (ER α +) positive breast cancer cells Tamoxifen has been widely used as an anti-cancer agent. Tamoxifen's mechanism of action is through ER. It binds to the receptor and leads to a conformational change which eventually prevents cancer cells proliferation and survival. In our current study, we aimed to investigate the combination of Tamoxifen with Vitamin D_3_ to test whether this combination will enhance the anti-cancer effect of Tamoxifen on breast cancer cells *in vitro*. Vitamin D_3_ has sterol structure and this property enables it to act similar to hormones. Vitamin D Receptor (VDR) has been commonly found in different types of cancer cells including but not limited to breast and prostate cancer cells. Through this receptor Vitamin D_3_ acts as an anti-proliferative agent. We examined the proliferation rate, apoptosis and necrosis levels as well as cell cycle progression in MCF-7 breast cancer cell line in the presence of Vitamin D_3_ and Tamoxifen to compare the changes with the Tamoxifen treated group. Our results suggest that Tamoxifen was a more potent anti-cancer agent than Vitamin D_3_ or its combination with Vitamin D_3_ based on cell cycle arrest, apoptosis and cell proliferation levels. This effect in the apoptosis rate and cell cycle stage of the MCF-7 cells were in line with the changes in gene expression profile of *P53*, *BAX* and *BCL-2*. Our results suggest that Tamoxifen by itself is adequate enough and more potent than Vitamin D_3_ or its combination with Vitamin D_3_ as anti-cancer agent for the breast cancer cells *in vitro*.

## Introduction

Cancer has become one of the leading causes of death after cardiovascular diseases (Jemal et al., 2009[9]; Motawi et al., 2016[14]). Breast cancer has the highest incidence rate among women and 18 % of cancer related deaths are due to breast cancer (Goldhirsch et al., 2009[7]). There are genetic and environmental risk factors that have been defined and associated with the development of breast cancer (Higgins and Baselga, 2011[8]). Although there have been some chemotherapeutic approaches against breast cancer the efficiency of the treatment is not at the desired point (Clarke et al., 2003[5]).

Problems with chemotherapy applications can be listed as low efficacy and high incidence of side effects. Studies in the field either focus on the development of novel chemotherapeutic agents or combination therapies to increase the efficacy of chemotherapeutics in use (Wang et al., 2019[21]; Karagül et al., 2020[10]).

In case of breast cancer treatment Tamoxifen has been commonly used against estrogen receptor alpha positive (ERα+) cells (Yang et al., 2013[22]). Tamoxifen shows its activity by binding to the estrogen receptor and changing its conformation (Mills et al., 2018[12]). It activates P53 and protein kinase C (PKC) pathways to induce apoptosis in tumor cells (Motawi et al., 2016[14]). It is also known as selective estrogen receptor modulator and has been in use for the last thirty years in breast cancer patients as supportive treatment agent (Chan et al., 2020[4]). When resistance against estrogen develops, tamoxifen loses its effectiveness. 25 % of Tamoxifen treated patients struggle with the recurrence issues and in the late stages only 30-40 % of the ERα+ patients respond to the anti-estrogen treatment (Shazia et al., 2016[19]).

Due to efficacy problems, combination of Tamoxifen with different drug molecules have been tried to increase its activity against tumor cells (Samadi et al., 2014[18]).

In our study, we focused on Vitamin D_3_. This vitamin has sterol structure and due to this property, it has hormone like functions. It is soluble in fat and has important functions in calcium homeostasis hence in bone formation and strength. It exerts its activity through Vitamin D Receptor (VDR). Through this receptor, it regulates cell proliferation and differentation pathways. VDR is expressed on breast, prostate, thyroid, hemotopoietic tumor cells and retinoblastomas. Vitamin D_3_ decreases cell proliferation, induces apoptosis and cell cycle arrest at G_0_/G_1 _phase. Vitamin D_3_ and its metabolites have been used as anti-cancer agents in pre-clinical and clinical trials due to these properties.

In our current study we utilized from MCF7 breast cancer cell line to examine the effect of Tamoxifen and Vitamin D_3 _on the cell cycle and apoptosis. There are studies combining these two molecules against breast cancer cells and they support an anti-proliferative activity for the combination. First time to our knowledge by our current study the effect of this combination therapy is examined in terms of changes in the rate of apoptosis and cell cycle progression. Our results suggest that depending on the time point the combination therapy had differential effects on breast cancer cell rates of apoptosis, necrosis and cell cycle arrest. Tamoxifen was more potent by itself as anti-cancer agent on the *in vitro *treated breast cancer cells compared to Vitamin D_3_ or its combination with Vitamin D_3_.

## Materials and Methods

### Cell culture

MCF-7 cells were obtained from American Type Culture Collection (ATCC^®^ HTB-22^™^. Manassas. VA. USA). These cells were cultured in RPMI medium (Catalog number: 11875093, Thermo Fisher Scientific, Inc., Waltham. MA. USA) supplemented with 10 % fetal bovine serum, penicillin/streptomycin, and amphotericine-B 1 %. Cell culture was performed at 37 °C in 5 % CO_2_ incubator. A preliminary study experiment was conducted to determine the dose of tamoxifen (SIGMA T5648-1G, Sigma-Aldrich, Merck) (10 μM, 20 μM, 40 μM and 60 μM) and vit D_3_ (Cayman Chemical) (10 nM, 50 nM, 75 nM, 100 nM, 125 nM, 200 nM, 500 nM and 1 µM). A combination study was carried out after the preliminary work experiment. MCF-7 cells without drugs or vitamins were used as control. MCF-7 cells were incubated with Tamoxifen (10 and 20 µM), vitamin D (50 and 100 nM) and combinations of both (Combination 1: TAM 10 μM + vit D 50 nM, Combination 2: TAM 10 μM + vit D 100 nM, Combination 3: TAM 20 μM + vit D 50 nM and Combination 4: TAM 20 μM + vit D 100 nM) for 72 h. 

### xCELLigence real-time cell analysis

The xCELLigence system (Real-Time Cell Analyzer (RTCA) Dual Plate (DP) (Roche Diagnostics GmbH, Penzberg) was used for real-time monitoring of cell viability without labeling on the cells. 3x10^4^ cells were seeded in each well of the E plate and the cell proliferation curve was monitored for 24 hours and after that, different Tamoxifen, vitamin D_3_ and their combinations were added to the E-plate systems and they were monitored in real time for 72 hours. Cell index (CI) value was automatically measured every hour for each well with RTCA Software (1.2.1).

### Detection of apoptotic cells in breast cancer cells by flow cytometry

In apoptotic cells, translocation of Phospholipid phosphatidylserine (PS) from inside to outside of the plasma membrane causes PS to be exposed to the external environment. In apoptotic cells, Annexin V is a phospholipid-binding protein for PS and binds to the surface of these cells due to PS exchange. Propidium iodide (PI) cannot cross the cell membrane due to membrane integrity in living cells. When the cell is damaged or dies, PI enters the cytoplasm and binds to DNA. MCF-7 cell density was adjusted to 1x10^6^ cells/ml and incubated in a 6-well plate. Cells were divided into different concentration groups of Tamoxifen (10 µM. 20 µM), vit D_3_ (50 nM and 100 nM), blank control group and combinations of both both (Combination 1: TAM 10 μM + vit D 50 nM, Combination 2: TAM 10 μM + vit D 100 nM, Combination 3: TAM 20 μM + vit D 50 nM and Combination 4: TAM 20 μM + vit D 100 nM) groups. After 48 and 72 h tamoxifen, vitamin and combination administration, the cells were removed and stained according to the manufacturer's recommendations (BioLegend's APC Annexin V Apoptosis Detection Kit with propidium iodide (PI) (cat. no. 640932 BioLegend. San Diego, CA). The cells were washed and then Annexin and PI staining solution were added and incubated in the dark for 15 minutes. The binding buffer was added and the percentages of apoptosis were analyzed on the BD FACSAria™ III flow cytometer (BD Biosciences. Bedford, MA, USA) using FACS Diva Software.

### Determination of cell cycle stages by flow cytometry

The cell cycle kit (BD 340242) works on the principle of dissolving lipids in the cell membrane with the help of detergents. Removing the cytoskeleton and nuclear proteins with the help of trypsin and breaking down cellular RNA by enzymes. This kit was used to determine cells with abnormal DNA and cell cycle phase distributions in the breast cancer cell line. MCF-7 cells were seeded at 1×10^6^ in 6 well plate and incubated for 24 h. After incubation, the media was removed and the cells were treated with different concentrations of TAM and vit D_3 _for 48 and 72 h. MCF-7 cells were then trypsinized and centrifuged at 500 g for 5 min. All supernatant was discarded, and Solution A (Trypsin Buffer) and Solution B were added and incubated at room temperature for 10 minutes. After then cold solution C was added. For cell cycle analysis, cells were stained with solution A (Trypsin Buffer), B (Trypsin inhibitor and RNase buffer) and C (Propidium Iodide Stain Solution) for 10 min and detection via a BD FACSAria™ III flow cytometer (BD Biosciences. Bedford, MA, USA) using FACS Diva Software and ModFit LT™.

### Effect of Tamoxifen, Vitamin D_3_ and both combinations on apoptosis regulating gene expression (P53, BAX and BCL-2) by quantitative real-time reverse transcription polymerase chain reaction (RT-qPCR) of MCF-7 cells

MCF-7 cell lines were cultured in 6-well plates and exposed to Tamoxifen, Vitamin D_3_ and both combination for 48 and 72 h. After incubation total RNA from MCF-7 cells was isolated using the high pure RNA isolation kit (cat. no. 1828665; Roche Life Science, Mannheim, Germany) as indicated by the producer's guidelines. Extracted RNAs were determined using a CapitalBio NanoQ™ spectrophotometer (CapitalBio Technology, China). Then cDNA was synthesized from total RNA using cDNA kit (cat. no. 4896866; Roche Life Science, Mannheim, Germany). Amplification reactions were established using LightCycler 480 PCR Master Mix (cat. No. 04707494001; Roche Life Science. Mannheim. Germany). The MCF-7 samples were incubated to 95 °C for 10 seconds for the first transcription of cDNA, followed by amplification for 45 cycles at 95 °C for 10 minutes, 60 °C for 30 seconds, and 72 °C for 1 minute. P53, BCL-2 and BAX mRNA expression values in accordance to the comparative CT method for quantitation of gene expression in MCF-7 samples are shown. Beta-actin (ACTB levels) RNA levels were used as normalization control in qRT-PCR (Livak and Schmittgen, 2001[11]). The expression of apoptosis-related genes (P53, BCL-2, BAX) (Table 1(Tab. 1)) was measured by qRT-PCR Lightcycler 480 II (Roche Life Science, Mannheim, Germany). 

### Statistical analysis

GraphPad Prism Version 5 was used for plotting the graphs and conducting the statistical analysis. Sample number (N) was at least three for each group. Student t test was applied to draw the significance in the difference (N=3; *p<0.001, **p<0.0005, ***p<0.0001).

## Results

### Anti-proliferative activities of Tamoxifen and Vitamin D_3 _was confirmed by xCELLigence system

In order to determine the optimum Tamoxifen and Vitamin D_3_ concentrations xCELLigence system was utilized. The system enables real time data for the cell proliferation. Based on the results presented in Supplementary Figures 1 and 2, the optimum test concentrations around IC_50_ values of Tamoxifen and Vitamin D_3_ were determined as 10 uM and 20 uM; 50 nM and 100 nM respectively (Supplementary Tables 1 and 2).

### Anti-proliferative activity of Tamoxifen, Tamoxifen and Vitamin D_3_ combination

xCELLigence system was used to set the following groups: Control untreated MCF-7 cells. Tamoxifen 10 uM, Tamoxifen 20 uM, Vitamin D_3_ 50 nM, Vitamin D_3_ 100 nM, combination of tamoxifen and vitamin D (Combination 1: TAM 10 μM + vit D 50 nM, Combination 2: TAM 10 μM + vit D 100 nM, Combination 3: TAM 20 μM + vit D 50 nM and Combination 4: TAM 20 μM + vit D 100nM) treated groups. The results were similar for all time points (24, 48 and 72 hours) and 20 µM Tamoxifen had the highest potency compared to the rest of the groups (Figure 3 and Table 4). xCELLigence results suggested that although combination therapy was effective to decrease the proliferation rate of MCF-7 cells, it was not as effective as 20 uM of Tamoxifen alone (Supplementary Figure 3). Combination of Vitamin D_3_ with the same concentration of Tamoxifen did not have a synergistic effect. Instead, the combination resulted in decrease in anti-proliferative activity of 20 uM of Tamoxifen (Table 2(Tab. 2)). Cell proliferation index of treatment of MCF-7 cells with Vit D_3_ administered at different concentrations (10 nM, 50 nM, 75 nM, 100 nM, 125 nM, 200 nM 500 nM and 1 µM).

### Tamoxifen was more effective than its combination with Vitamin D_3_ or Vitamin D_3_ alone for the induction of apoptosis in MCF-7 cells

**Figure 1:**
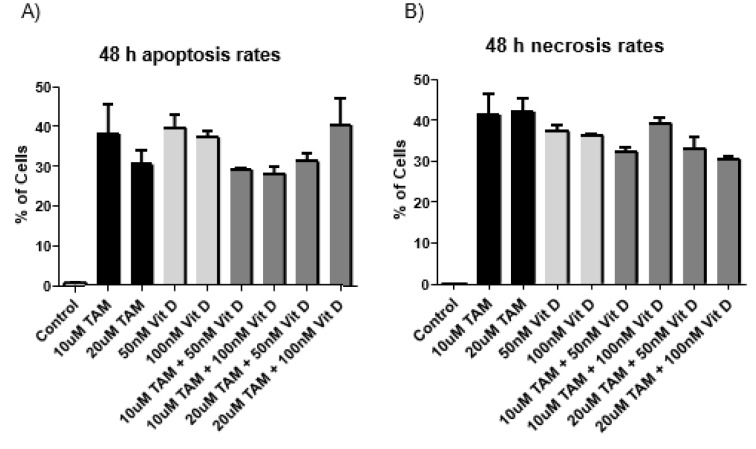
Apoptosis and necrosis levels of the MCF-7 cells after Tamoxifen 10 uM, Tamoxifen 20 uM, Vitamin D_3_ 50 nM, Vitamin D_3_ 100 nM and combination of both (Combination 1: TAM 10 μM + vit D 50 nM, Combination 2: TAM 10 μM + vit D 100 nM, Combination 3: TAM 20 μM + vit D 50 nM and Combination 4: TAM 20 μM + vit D 100 nM). The cells were incubated for 48 hours. 1x10^6^ cells/mL were plated in 6 well plates and after treatment with appropriate compounds and incubation of cells for 48 hours. The samples were stained with Annexin V and PI dye and analyzed by flow cytometry. The results were plotted as graphs and a representative flow cytometry image is given beneath the graphs.

**Figure 2:**
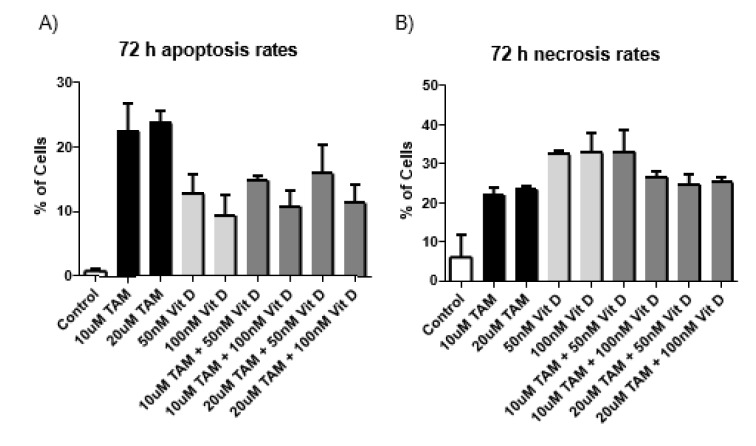
Apoptosis and necrosis levels of MCF-7 cells after Tamoxifen 10 uM, Tamoxifen 20 uM, Vitamin D_3_ 50 nM, Vitamin D_3_ 100 nM and combination of both (Combination 1: TAM 10 μM + vit D 50 nM, Combination 2: TAM 10 μM + vit D 100 nM, Combination 3: TAM 20 μM + vit D 50 nM and Combination 4: TAM 20 μM + vit D 100 nM). The cells were incubated for 72 hours. 1x10^6^ cells/mL were plated in 6 well plates and after treatment with appropriate compounds and incubation of cells for 72 hours. The samples were stained with Annexin V and PI dye and analyzed by flow cytometry. The results were plotted as graphs and a representative flow cytometry image is given beneath the graphs.

Flow cytometry analysis was conducted after 48 and 72 h treatment of MCF-7 cells with Tamoxifen 10 uM, Tamoxifen 20 uM, Vitamin D_3_ 50 nM, Vitamin D_3_ 100 nM and combination of both (Combination 1: TAM 10 μM + vit D 50 nM, Combination 2: TAM 10 μM + vit D 100 nM, Combination 3: TAM 20 μM + vit D 50 nM and Combination 4: TAM 20 μM + vit D 100 nM). When the data from both time points were analyzed, Tamoxifen induced higher rates of apoptosis on MCF-7 cells compared to the rest of the groups (Tables 3(Tab. 3) and 4(Tab. 4)). These results were in line with the xCelligence results. Moreover, there was no meaningful difference between the groups for the induction of necrosis (Figures 1(Fig. 1) and 2(Fig. 2)), (Supplementary Figures 4-5).

### Tamoxifen, Vitamin D_3_ and combination of both resulted in similar levels of cell cycle arrest at G_1_

**Figure 3:**
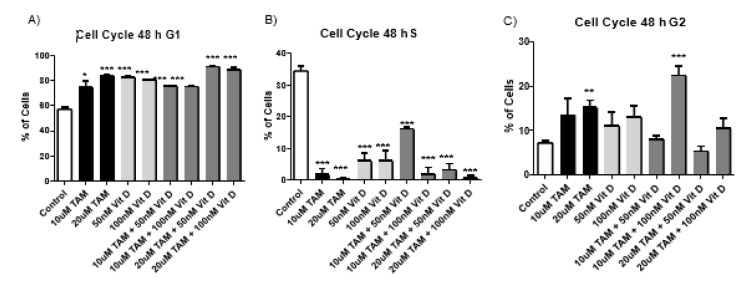
Cells in different stages of cell cycle after 10 uM Tamoxifen, 20 uM Tamoxifen, 50 nM Vitamin D_3_, 100 nM Vitamin D_3_ and combination of both (Combination 1: TAM 10 μM + vit D 50 nM, Combination 2: TAM 10 μM + vit D 100 nM, Combination 3: TAM 20 μM + vit D 50 nM and Combination 4: TAM 20 μM + vit D 100 nM). The MCF-7 cells were incubated for 48 hours; 1x10^6^ cells/mL were plated in 6 well plates and after treatment with appropriate compounds and incubation of cells for 48 hours, the samples were stained with PI dye and analyzed by flow cytometry. The results were plotted as graphs and a representative flow cytometry image is given beneath the graphs (N=3; *p<0.001,** p<0.0005, ***p<0.0001)

**Figure 4:**
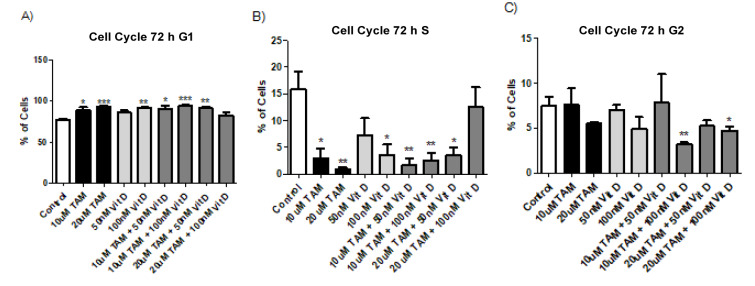
Cells in different stages of cell cycle after 10 uM Tamoxifen, 20 uM Tamoxifen, 50 nM Vitamin D_3_, 100 nM Vitamin D_3_ and combination of both (Combination 1: TAM 10 μM + vit D 50 nM, Combination 2: TAM 10 μM + vit D 100 nM, Combination 3: TAM 20 μM + vit D 50 nM and Combination 4: TAM 20 μM + vit D 100 nM). The MCF-7 cells were incubated for 72 hours; 1x10^6^ cells/mL were plated in 6 well plates and after treatment with appropriate compounds and incubation of cells for 72 hours, the samples were stained with PI dye and analyzed by flow cytometry. The results were plotted as graphs and a representative flow cytometry image is given beneath the graphs (N=3; *p<0.001, ** p<0.0005, ***p<0.0001).

Flow cytometry analysis was conducted for PI staining of the DNA content, after 48 and 72 hours treatment of MCF-7 cells with Tamoxifen 10 uM, Tamoxifen 20 uM, Vitamin D_3_ 50 nM, Vitamin D_3_ 100 nM and combination of both (Combination 1: TAM 10 μM + vit D 50 nM, Combination 2: TAM 10 μM + vit D 100 nM, Combination 3: TAM 20 μM + vit D 50 nM and Combination 4: TAM 20 μM + vit D 100 nM). At 48 and 72 hours time points the results were similar compared to control groups for all treatment groups; the percentage of cells in S phase of the cell cycle was significantly lower whereas the percentage of cells in G1 phase of the cells cycle was higher (Tables 5(Tab. 5) and 6(Tab. 6)). These results suggest that Tamoxifen, Vitamin D3 and combination of Tamoxifen and Vitamin D_3_ similarly affected the cell cycle progression and led to cell cycle arrest at G_1 _phase (Figures 3(Fig. 3) and 4(Fig. 4)) (Supplementary Figures 6 and 7).

### Tamoxifen, Vitamin D_3_ and their combination exerted their anti-cancer activity by similarly decreasing gene expression levels for P53 and BCL2. The proteins were involved in cell cycle progression and apoptosis respectively

**Figure 5:**
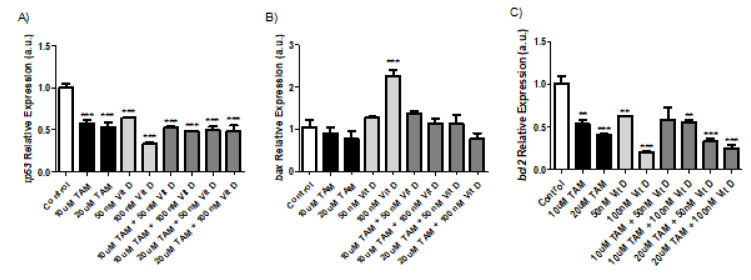
*P53, BCL2 and BAX genes *expression level for MCF-7 cells treated with 10 uM Tamoxifen, 20 uM Tamoxifen, 50 nM Vitamin D_3_, 100 nM Vitamin D_3_, and combination of both (Combination 1: TAM 10 μM + vit D 50 nM, Combination 2: TAM 10 μM + vit D 100 nM, Combination 3: TAM 20 μM + vit D 50 nM and Combination 4: TAM 20 μM + vit D 100 nM) for 72 hours. The cells were incubated for 72 hours 1x10^6^ cells/mL were plated in 6 well plates and after treatment with appropriate compounds and incubation of cells for 72 hours, the cDNAs were generated from each group of cells to determine the gene expression levels (N=3; *p<0.001, ** p<0.0005, ***p<0.0001).

Q-RT-PCR was done for the cDNA samples from MCF-7 cells that were treated for 72 hours with the following compounds: 10 µM Tamoxifen. 20 µM Tamoxifen, 50 nM Vitamin D_3_, 100 nM Vitamin D_3_ and combination of both (Combination 1: TAM 10 μM + vit D 50 nM, Combination 2: TAM 10 μM + vit D 100 nM, Combination 3: TAM 20 μM + vit D 50 nM and Combination 4: TAM 20 μM + vit D 100 nM). P53 plays an important role in the arrest of cell cycle progression as a tumor supressor and its expression is lowered in tumor cells. Compared to control groups Tamoxifen, Vitamin D_3_ and their combination therapy lead to significant decreases in P53 expression levels. These compounds either separately or in combination had similar levels (Figure 5(Fig. 5)). BCL-2 is known to be an anti-apoptotic protein and according to our results, its expression levels were significantly decreased in treated groups compared to the control group. This trend was in line with increasing apoptosis levels (Figures 1(Fig. 1), 2(Fig. 2) and 5(Fig. 5)). BAX protein can act as anti- or pro-apoptotic depending on its dimerized couple. BAX gene expression levels did not change compared to control group after the treatments (Figure 5(Fig. 5)). Both for BCL2 and BAX expression levels, Tamoxifen or Vitamin D_3_ or their combinations had similar effects (Figure 5(Fig. 5)).

## Discussion

Breast cancer has the highest incidence rate among women throughout the world and treatment options for this cancer are limited (Su et al., 2008[20]; Colston et al., 1981[6]). Approved and clinically acclaimed chemotherapy applications suffer from low efficacy as well as unwanted side effects (Pondé et al., 2019[16]). Other than developing novel chemotherapy agents with higher efficiencies and lower side effects, combination therapy stands as a plausible application to eradicate the tumor. Another advantage of combination therapy is that the stages of drug development and application procedures would be bypassed since the drug molecules that are already in use are tried to create a synergistic effect.

Tamoxifen is a commonly used chemotherapeutic against estrogen receptor alpha positive tumor cells (Yang et al., 2013[22]). It leads to a change in the conformation of the ER receptor and induces apoptosis of the tumor cells by activating P53 and PKC pathways (Mohamed et al., 2020[13]). Resistance agains the estrogen is the main problem with the Tamoxifen treatments and generating a combination with a drug molecule that targets different pathways might overcome the issue of estrogen resistance. Estrogen resistance develops by time after the treatment and targetting different pathways would most likely prevent the development of the estrogen resistance and would make Tamoxifen effective in the treatment regimens (Mohamed et al., 2020[13]; Motawi et al., 2016[14]).

In our current study we chose Vitamin D_3 _as part of the combination therapy together with Tamoxifen against breast cancer cell line. Its receptor, VDR, regulates the cell proliferation and differentiation pathways. This receptor is expressed in different types of cancer cells including but not limited to breast, prostate, thyroid and hemotopoietic tumors. Studies suggest that the mechanism of action of Vitamin D_3 _is to block the cell proliferation (Yingyu et al., 2010[23], Abe-Hashimoto et al., 1993[1]; Axanova et al., 2010[3]), inducing cell death and causing cell cycle arrest at G_0_/G_1_ phase (Zheng et al., 2019[25]).

Metabolites of Vitamin D_3 _are also used on clinical settings to enhance the activities of chemotherapeutics. Therefore, we hypothesized that since Tamoxifen works through ER and Vitamin D_3_ works through PKC pathway their combination might have synergistics anti-cancer effects. Previous studies suggest that their combination led to higher anti-proliferative activity on breast cancer cells compared to their utilization by themselves (Yingyu et al., 2010[23]; Abe-Hashimoto et al., 1993[1]). Our results are not in line with those studies since Tamoxifen's higher concentration was the most effective in decreasing the proliferation of MCF-7 cells compared to Vitamin D_3_ or its combination with Vitamin D_3_. Instead of having a synergistic effect, their combination actually decreased the activity of Tamoxifen (Table 2(Tab. 2)) (Supplementary Figure 3). At the cell proliferation level their combinations blocked. Moreover, the apoptotic and necrotic cell levels were comparable between the groups and in line with the cell proliferation results, higher concentration of Tamoxifen was the most effective treatment (Tables 3(Tab. 3) and 4(Tab. 4)). Combination therapy was not as effective as Tamoxifen's higher concentration (Figures 1(Fig. 1) and 2(Fig. 2)) (Supplementary Figures 4 and 5). Furthermore, all the treatment regimens similarly blocked the cell cycle progression at G1 phase (Tables 5(Tab. 5) and 6(Tab. 6)). Different concentrations of Tamoxifen, Vitamin D_3 _and their combinations similarly blocked the cell cycle progression towards S phase (Figures 3(Fig. 3) and 4(Fig. 4)) (Supplementary Figures 6 and 7).

In order to decipher the activities of Tamoxifen, Vitamin D_3_ and their combinations at gene expression level, three genes expression's were measured by Q-RT-PCR. P53 is a tumor suppressor whose expression decreases in tumor cells to go through cell cycle and proliferate (Zheng et al., 2019[25]). Previous studies suggest that Vitamin D_3_ or melatonin and Vitamin D_3_ combination exerted its anti-proliferative effect on MCF-7 cells by upregulating P53 expression both at gene and protein level (Raman et al., 2018[17]). Our results suggest that Vitamin D_3_ or its combination with Tamoxifen led to a significant decrease in gene expression levels of P53 compared to control untreated groups (Figure 5(Fig. 5)). The reason behind this discrepancy between that study and our study is unknown. There are also studies suggesting that Vitamin D_3_ exerts its activity on tumor cells depending on P53 expression level. Those studies suggest that the mutated form of P53 alters Vitamin D's activity to make it anti-apoptotic instead of pro-apoptotic (Perry et al., 2010[15]). Another study suggests that Tamoxifen did not change gene expression levels of BAX or P53, our results overlap for BAX expression levels but not for P53 (Zhang et al., 1999[24]). P53 expression levels significantly dropped after Tamoxifen, Vitamin D_3_ or their combination treatment. There were no differences between the treatment groups. BAX is another protein that is involved in apoptosis. Depending on its dimerization partner it can either act as anti- or pro-apoptotic. In all treated groups BAX expression was similar to that in the control groups. BCL-2 is an anti-apoptotic protein and its gene expression levels were significantly lower in groups treated with Tamoxifen, Vitamin D_3_ and their combinations compared to control groups. This decrease was in line with previous findings (Alamro et al., 2021[2]). Discrepencies between our results and previously published work might stem from differences in experimental protocols. There are studies conflicting with each other in the literature as well (Perry et al., 2010[15]; Zhang et al., 1999[24]; Alamro et al., 2021[2]). Our results and their findings suggest that this field requires further investigations. Overall, Tamoxifen and Vitamin D_3_ did not exert a synergistic anti-cancer effect on breast cancer cells. Tamoxifen was more potent by itself at its higher concentrations compared to Vitamin D_3_ alone or its combination with Vitamin D_3_. The mechanism of action was to block proliferation, cell cycle progression and induction of apoptosis. Expression levels of anti-apoptotic BCL-2 gene was lowered at comparable levels after the treatment with Tamoxifen, Vitamin D_3_ and their combinations, P53 tumor suppressor's expression levels were also lower in those treatment groups. Most probably the mutated form of P53 was downregulated to prevent cell cycle progression and induces apoptosis in the breast cancer cells since there was a cell cycle arrest and increased apoptosis in the cell groups that were treated with Tamoxifen, Vitamin D_3_ and their combinations. It should be emphasized that we used only one cell line in this study. More studies with different ER+ and ER-cell lines should be conducted for generalization of our results. Depending on the cell line type the results may also conflict or overlap each other. Therefore, further investigation of Vitamin D_3_ and Tamoxifen would be informative for the field. 

In conclusion, further studies are needed to determine the most compatible compound for combination therapy with Tamoxifen. Our results suggest that Vitamin D_3_ is not a good candidate for combination with Tamoxifen against breast cancer cells. In our future studies, we will focus on other FDA approved drug candidates for their possible usage against breast cancer cells in combination with Tamoxifen.

## Notes

Derya Yetkin and Furkan Ayaz (Mersin University, Department of Biotechnology, Faculty of Arts and Science, 33110, Mersin, Turkey; Tel: 00 90-324-3610000, E-mail: furkanayaz@mersin.edu.tr) contributed equally as corresponding author.

## Acknowledgements

This work was partially supported by a Scientific Research Project (BAP) grant BAP-2015-TP3-1293.

## Supplementary Material

Supplementary information

## Figures and Tables

**Table 1:**
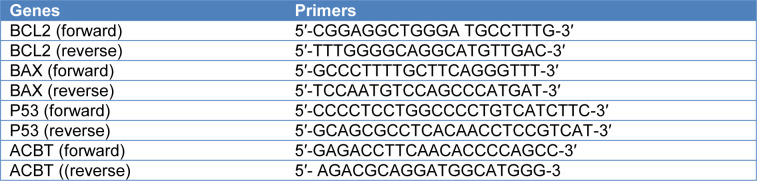
Primer sets for each gene

**Table 2:**
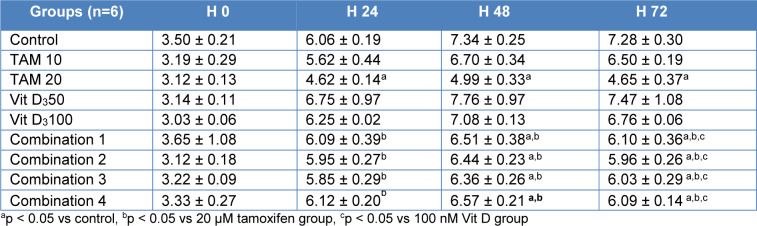
Cell proliferation index of MCF-7 cells treatment with Tamoxifen (10 and 20 μM), vitamin D_3_ (50 and 100 nM) and combinations of both (Combination 1: TAM 10 μM + vit D 50 nM, Combination 2: TAM 10 μM + vit D 100 nM, Combination 3: TAM 20 μM + vit D 50 nM and Combination 4: TAM 20 μM + vit D 100 nM)

**Table 3:**
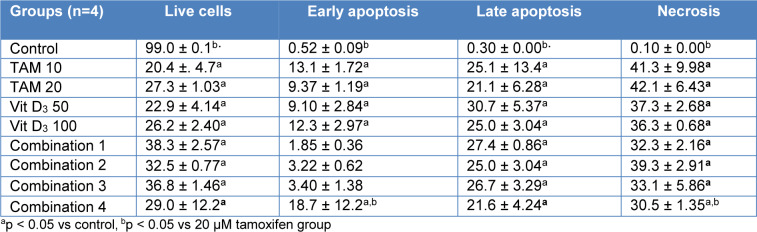
Evaluation of apoptosis/necrosis in MCF-7 cells treated with the combination of Tamoxifen and vitamin D_3_ for 48 hours using the Annexin V-PI assay in flow cytometry

**Table 4:**
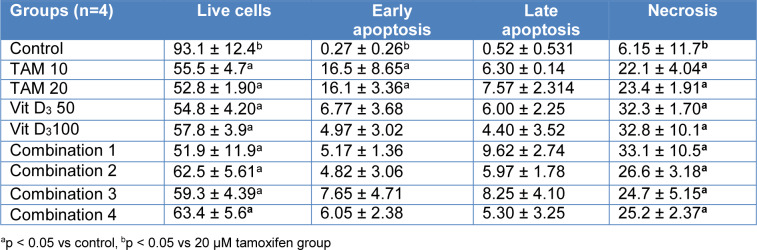
Evaluation of apoptosis / necrosis in MCF-7 cells treated with the combination of Tamoxifen and vitamin D_3_ for 72 hours using the Annexin V-PI assay in flow cytometry

**Table 5:**
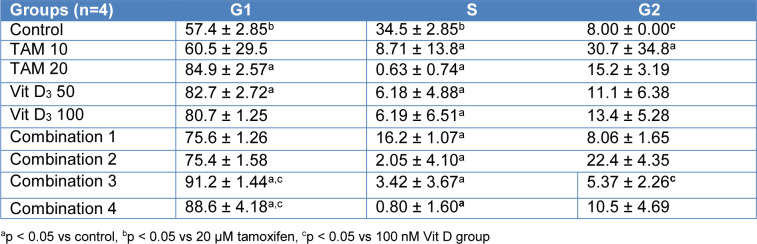
Flow cytometry analysis for cell cycle distribution of MCF-7 cells treatment with Tamoxifen, vitamin D and combinations of both for 48 h

**Table 6:**
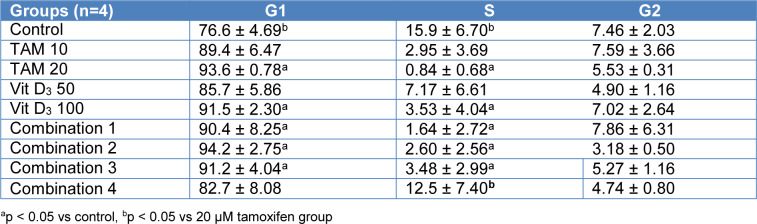
Flow cytometry analysis for cell cycle distribution of MCF-7 cells treatment with Tamoxifen, vitamin D_3_ and combinations of both for 72 h
