# Supplementary material for: Antiproliferative activity of Tamoxifen, Vitamin D3 and their concomitant treatment
Source: EXCLI J. 2021 Sep 21;20:1394–406. doi: 10.17179/excli2021-3989 (PMC8564918; doi:10.17179/excli2021-3989)
Supplement: Supplementary information [file EXCLI-20-1394-s-001.pdf]

EXCLI Journal 2021;20:1394-1406 – ISSN 1611-2156
Received: June 14, 2021, accepted: August 24, 2021, published: September 21, 2021

Supplementary information to:

Original article:

ANTIPROLIFERATIVE ACTIVITY OF TAMOXIFEN, VITAMIN D3 AND THEIR CONCOMITANT TREATMENT

Derya Yetkin[1*], Ebru Balli[2], Furkan Ayaz[3*]

[1] Mersin University, Advanced Technology Education Research and Application Center, 33110, Mersin, Turkey
[2] Mersin University, Department of Histology and Embryology, 33110 Mersin, Turkey
[3] Mersin University, Department of Biotechnology, Faculty of Arts and Science, 33110, Mersin, Turkey

* **Corresponding authors:** Furkan Ayaz, Mersin University, Department of Biotechnology, Faculty of Arts and Science, 33110, Mersin, Turkey. Tel: 00 90-324-3610000, E-mail: furkanayaz@mersin.edu.tr;
Derya Yetkin, Mersin University, Advanced Technology Education Research and Application Center, 33110, Mersin, Turkey. Tel: 00 90-324-3610000, E-mail: deryayetkin@mersin.edu.tr

http://dx.doi.org/10.17179/excli2021-3989

This is an Open Access article distributed under the terms of the Creative Commons Attribution License (http://creativecommons.org/licenses/by/4.0/).

EXCLI Journal 2021;20:1394-1406 – ISSN 1611-2156
Received: June 14, 2021, accepted: August 24, 2021, published: September 21, 2021

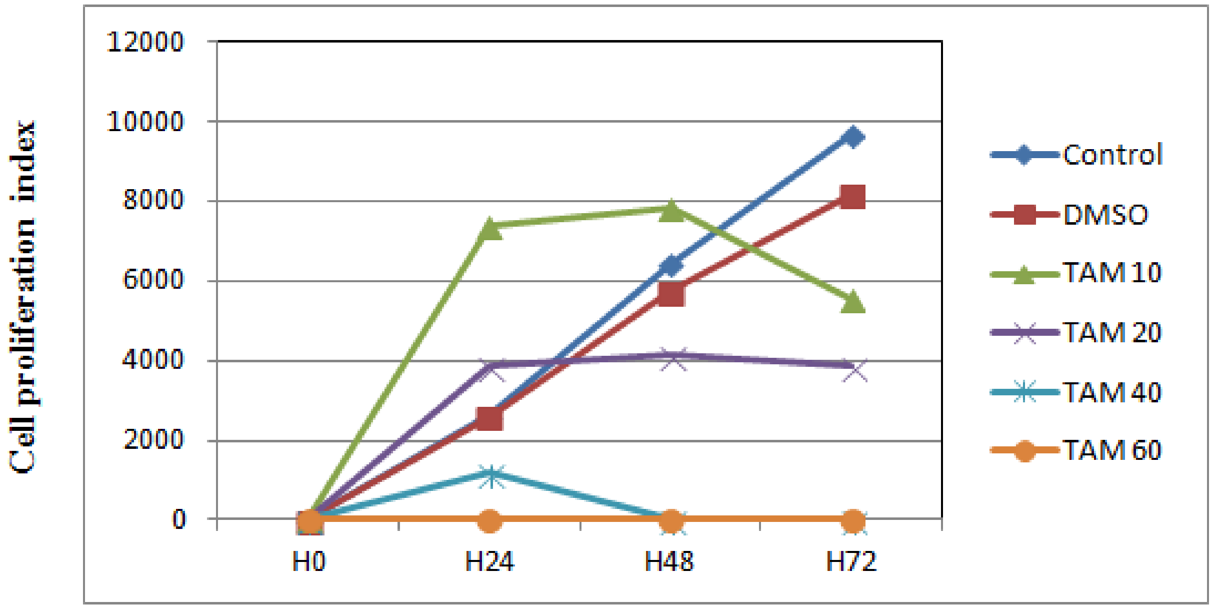

Supplementary Figure 1: Time dependent graph of cell index values of different tamoxifen concentrations in MCF-7 cells for 72 h calculated in RTCA Software 1.2.1.

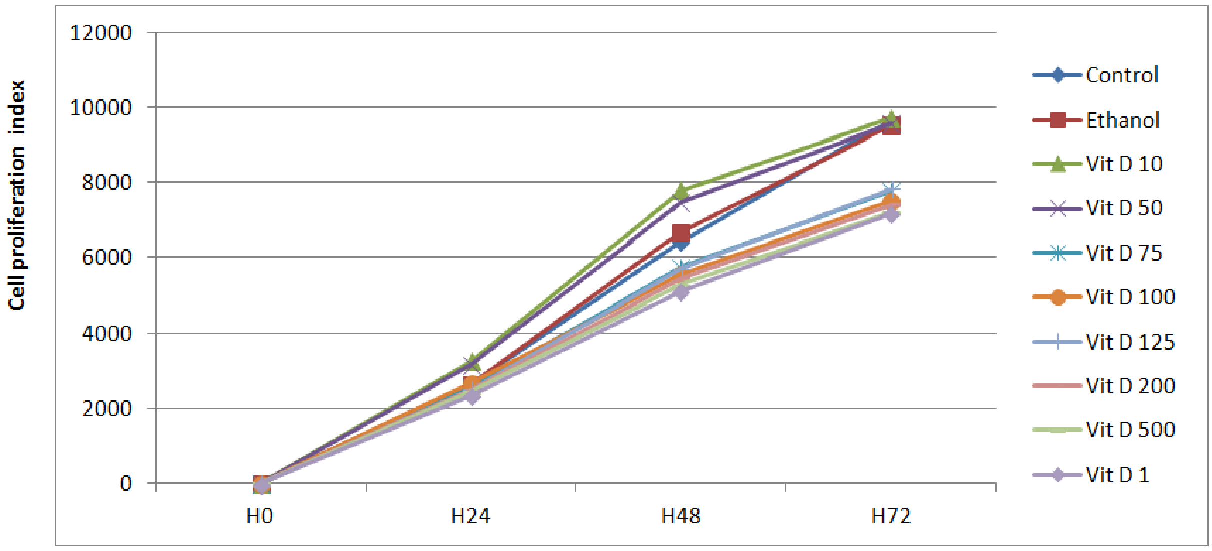

Supplementary Figure 2: Time dependent graph of cell index values of different Vitamin D_3 concentrations in MCF-7 cells for 72 h calculated in RTCA Software 1.2.1.

EXCLI Journal 2021;20:1394-1406 – ISSN 1611-2156
Received: June 14, 2021, accepted: August 24, 2021, published: September 21, 2021

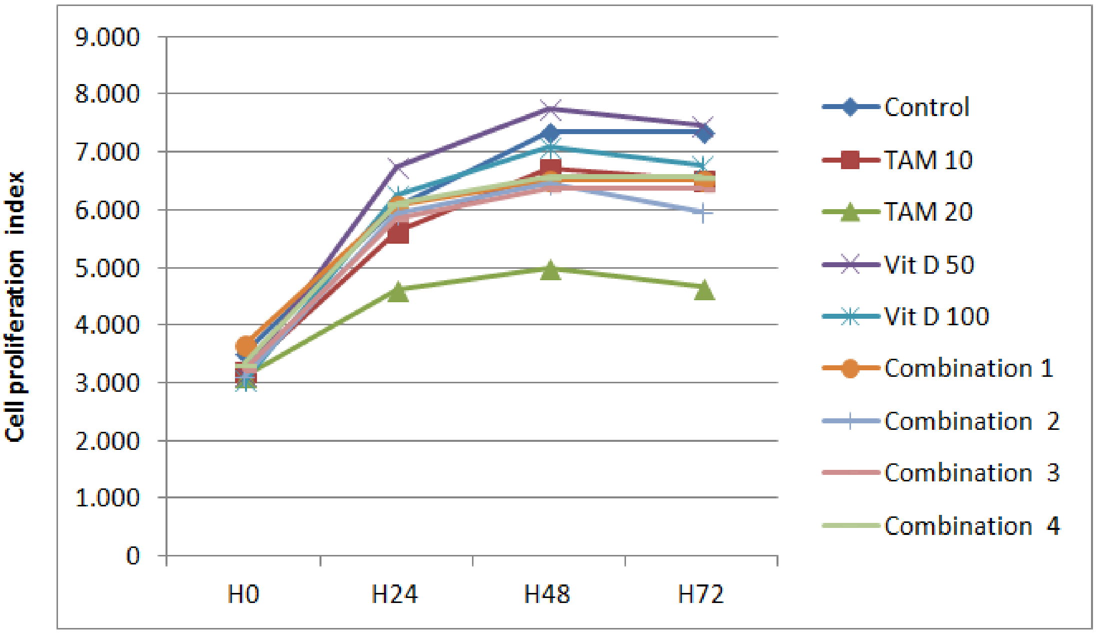

Supplementary Figure 3: Time dependent graph of cell index values of different concentration combinations of tamoxifen and Vitamin D_3 (Combination 1: TAM 10 μM + vit D 50 nM, Combination 2: TAM 10 μM + vit D 100 nM, Combination 3: TAM 20 μM + vit D 50 nM and Combination 4: TAM 20 μM + vit D 100 nM) in MCF-7 cells for 72 h calculated in RTCA Software 1.2.1.

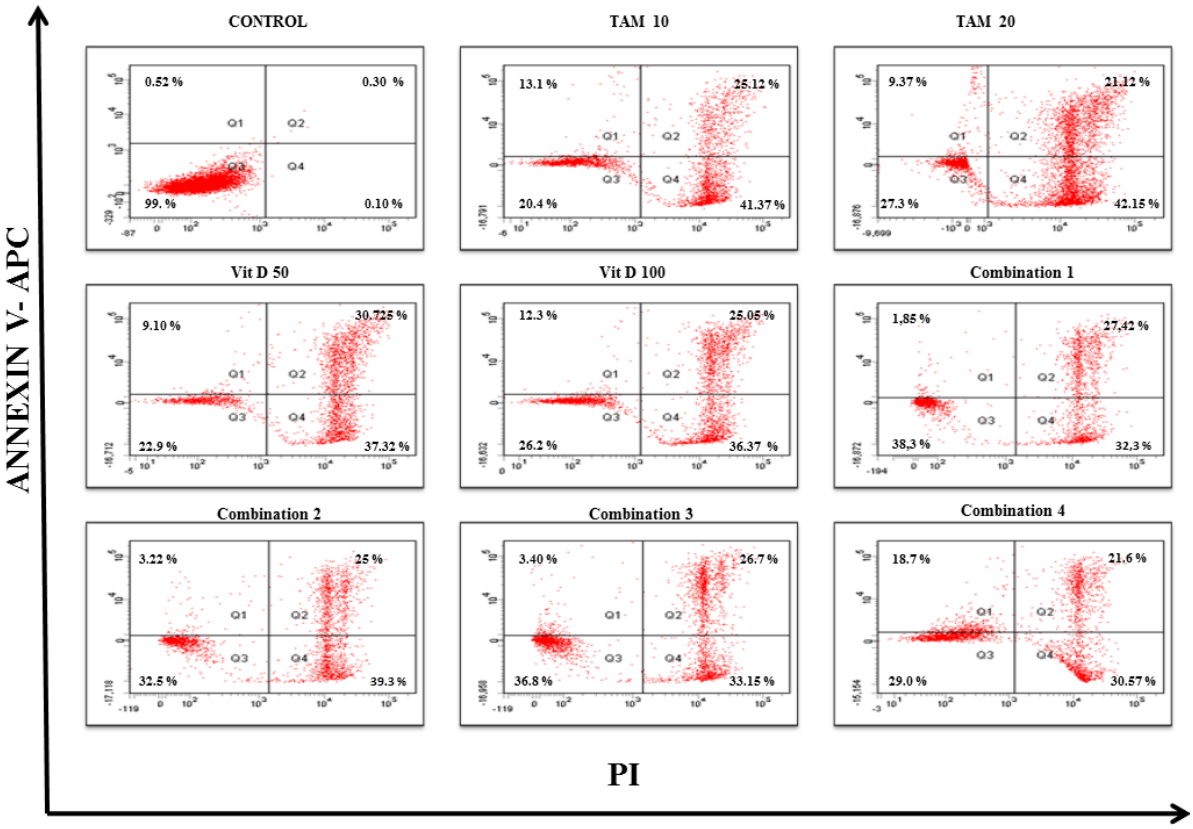

Supplementary Figure 4: Apoptosis and necrosis analysis of MCF-7 cells was evaluated in flow cytometry, after tamoxifen, vitamin D_3 and combination therapy for 48 h. Apoptosis data; live cells (Q3), early apoptosis (Q1), late apoptosis (Q2) and necrosis (Q4) populations were identified by plotting Annexin V staining intensity versus PI staining intensity.

EXCLI Journal 2021;20:1394-1406 – ISSN 1611-2156
Received: June 14, 2021, accepted: August 24, 2021, published: September 21, 2021

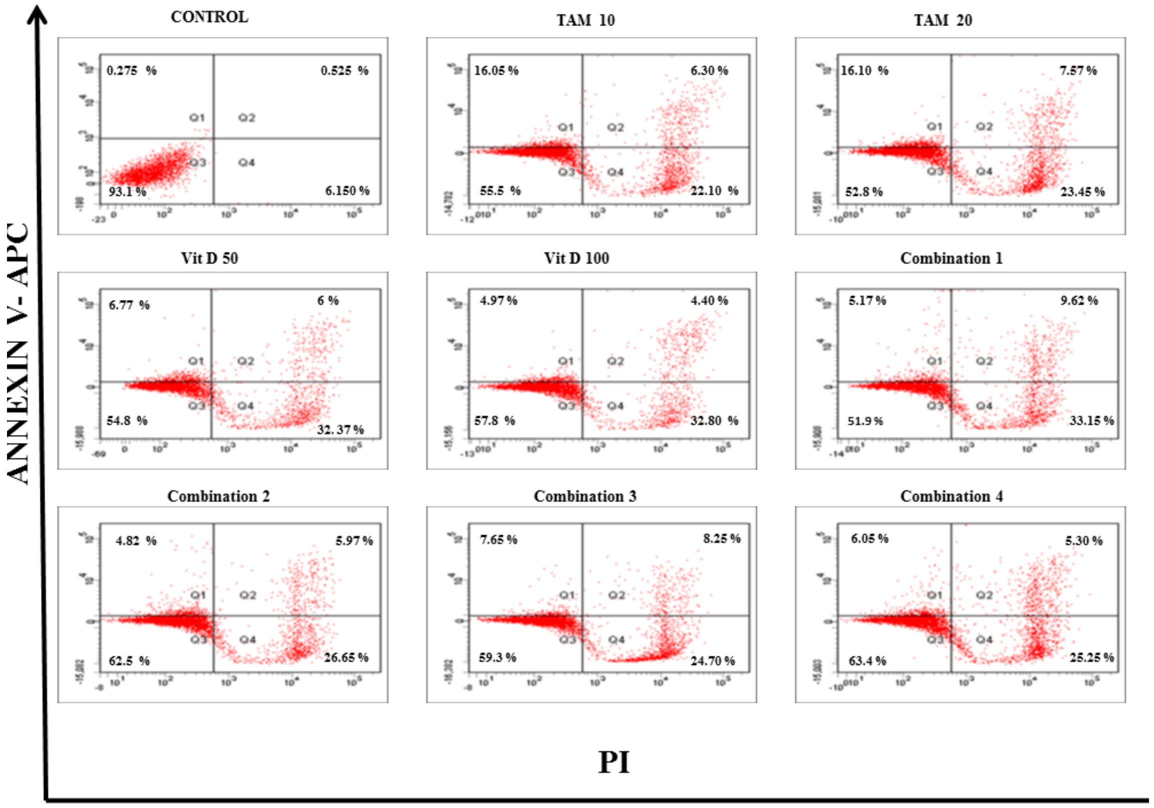

Supplementary Figure 5: Apoptosis and necrosis analysis of MCF-7 cells was evaluated in flow cytometry, after tamoxifen, vitamin D_3 and combination therapy for 72 h. Apoptosis data; live cells (Q3), early apoptosis (Q1), late apoptosis (Q2) and necrosis (Q4) populations were identified by plotting Annexin V staining intensity versus PI staining intensity.

EXCLI Journal 2021;20:1394-1406 – ISSN 1611-2156
Received: June 14, 2021, accepted: August 24, 2021, published: September 21, 2021

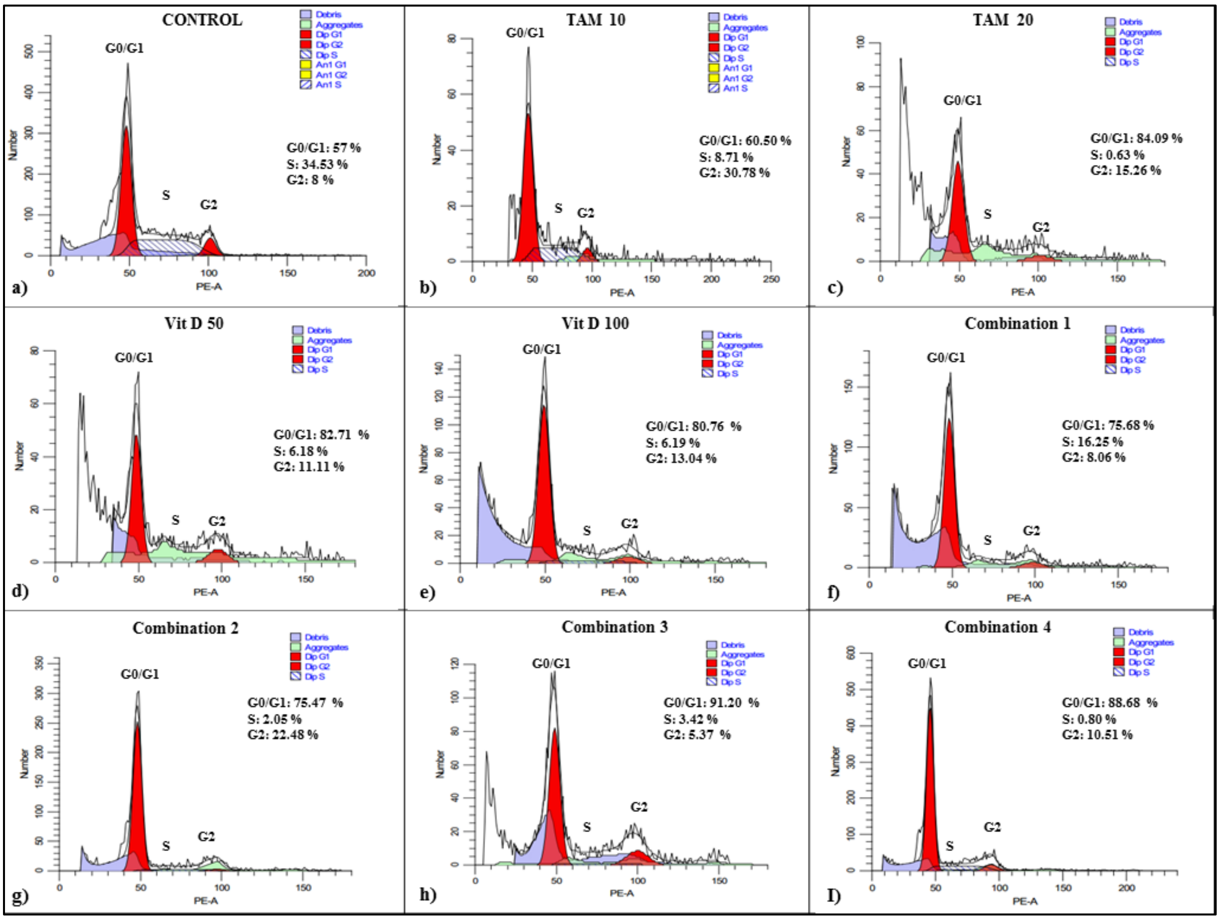

Supplementary Figure 6: Cell-cycle analysis by flow cytometry of MCF-7 cells treated with control (**a**), Tamoxifen (**b**: 10 µM; **c**: 20 µM), vitamin D_3 (**d**: 50 nM; **e**: 100 nM) and combination of both (**f**: Combination 1: TAM 10 µM + vit D 50 nM, **g**: Combination 2: TAM 10 µM + vit D 100 nM, **h**: Combination 3: TAM 20 µM + vit D 50 nM and **i**: Combination 4: TAM 20 µM + vit D 100 nM for 48 h.

EXCLI Journal 2021;20:1394-1406 – ISSN 1611-2156
Received: June 14, 2021, accepted: August 24, 2021, published: September 21, 2021

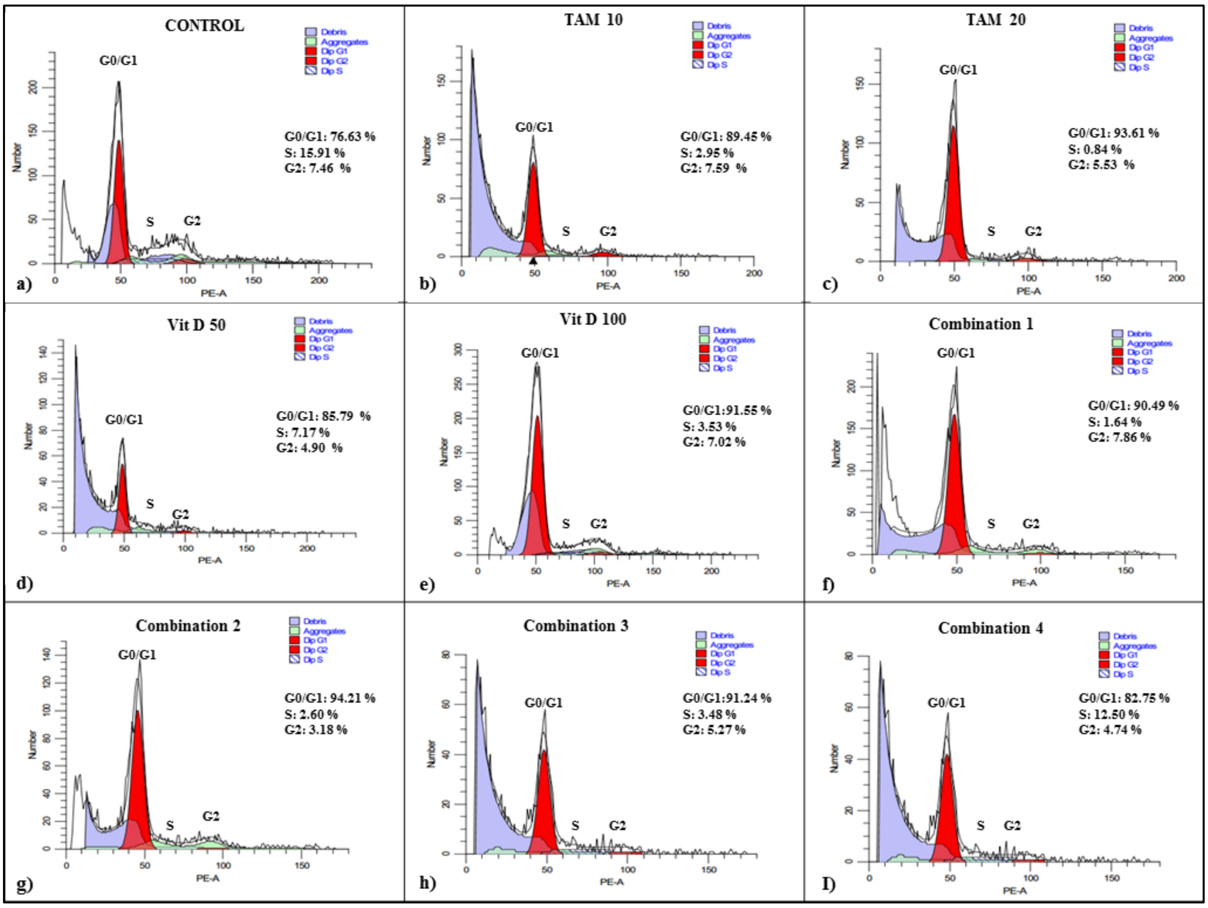

Supplementary Figure 7: Cell-cycle analysis by flow cytometry of MCF-7 cells treated with control (**a**), Tamoxifen (**b**: 10 µM; **c**: 20 µM), vitamin D_3 (**d**: 50 nM; **e**: 100 nM) and combination of both (**f**: Combination 1: TAM 10 µM + vit D 50 nM, **g**: Combination 2: TAM 10 µM + vit D 100 nM, **h**: Combination 3: TAM 20 µM + vit D 50 nM and **i**: Combination 4: TAM 20 µM + vit D 100 nM for 72 h.

EXCLI Journal 2021;20:1394-1406 – ISSN 1611-2156
Received: June 14, 2021, accepted: August 24, 2021, published: September 21, 2021

Supplementary Table 1: Average of cell proliferation index obtained between groups 24, 48 and 72 hours after tamoxifen treatment in MCF7 cells

Groups (n=4)	H 0	H 24	H 48	H 72
Control	0.772 ± 0.167	2.623 ± 0.546	6.445 ± 1.378	9.675 ± 0.789
DMSO	0.652 ± 0.435	2.600 ± 0.311	5.743 ± 0.745	8.165 ± 1.019
TAM 10	0.918 ± 0.125	7.350 ± 0.723	7.794 ± 0.520	5.531 ± 0.324[a]
TAM 20	0.888 ±0.101	3.863 ± 0.333[a]	4.121 ± 0.421[a]	3.854 ± 0.328[a]
TAM 40	0.811 ± 0.094	1.166 ± 0.114[a]	0.980 ± 0.084[a]	0.7189 ± 0.0911[a]
TAM 60	0.848 ± 0.136	0.725 ± 0.131[a]	0.998 ± 0.172[a]	0.6324 ± 0.094[a]

[a] p <0.05 compared to the control group

Supplementary Table 2: Cell proliferation index of treatment of MCF-7 cells with Vit D_3 administered at different concentrations

Groups (n=4)	H 0	H 24	H 48	H 72
Control	0.772 ± .167	2.623 ± .546	6.445 ± 1.378	9.675 ± .789
Ethanol	0.882 ± .010	2.623 ± .546	6.695 ± .936	9.549 ± 1.410
Vit D_3 10	0.926 ± .122	3.252 ± .567	7.791 ± 1.613	9.722 ± 1.418
Vit D_3 50	0.934 ± .056	3.165 ± .090	7.475± .896	9.576 ±. 987
Vit D_3 75	0.790 ± .112	2.573 ± .420	5.742 ± .938	7.794 ± .917[a]
Vit D_3 100	0.846± .101	2.660 ± .301	5.555 ± .478	7.512 ± .566[a]
Vit D_3 125	0.877 ± .066	2.503± .153	5.710 ± .344	7.829 ± .545[a]
Vit D_3 200	0.886 ± .0.53	2.476 ± .543	5.442 ± .348	7.427 ± .435[a]
Vit D_3 500	.879 ± .0.48	2.454 ± .087	5.288 ± .568	7.218 ± .384[a]
Vit D_3 1	.891 ± .0.72	2.335 ± .564	5.122 ± .479	7.195 ± .158[a]

[a]p< 0.05 is significantly different vs control with vitamin D groups
